# AMP-Activated Protein Kinase (AMPK)-Dependent Regulation of Renal Transport

**DOI:** 10.3390/ijms19113481

**Published:** 2018-11-06

**Authors:** Philipp Glosse, Michael Föller

**Affiliations:** 1 Institute of Agricultural and Nutritional Sciences, Martin Luther University Halle-Wittenberg, D-06120 Halle (Saale), Germany; philipp.glosse@landw.uni-halle.de; 2 Institute of Physiology, University of Hohenheim, D-70599 Stuttgart, Germany

**Keywords:** transporter, carrier, pump, membrane, energy deficiency

## Abstract

AMP-activated kinase (AMPK) is a serine/threonine kinase that is expressed in most cells and activated by a high cellular AMP/ATP ratio (indicating energy deficiency) or by Ca^2+^. In general, AMPK turns on energy-generating pathways (e.g., glucose uptake, glycolysis, fatty acid oxidation) and stops energy-consuming processes (e.g., lipogenesis, glycogenesis), thereby helping cells survive low energy states. The functional element of the kidney, the nephron, consists of the glomerulus, where the primary urine is filtered, and the proximal tubule, Henle’s loop, the distal tubule, and the collecting duct. In the tubular system of the kidney, the composition of primary urine is modified by the reabsorption and secretion of ions and molecules to yield final excreted urine. The underlying membrane transport processes are mainly energy-consuming (active transport) and in some cases passive. Since active transport accounts for a large part of the cell’s ATP demands, it is an important target for AMPK. Here, we review the AMPK-dependent regulation of membrane transport along nephron segments and discuss physiological and pathophysiological implications.

## 1. Introduction

The 5′-adenosine monophosphate (AMP)–activated protein kinase (AMPK) is a serine/threonine protein kinase that is evolutionarily conserved and functions as an intracellular energy sensor in mammalian cells [1,2,3,4,5]. It is a central regulator of energy homeostasis and affects many important cellular functions including growth, differentiation, autophagy, and metabolism [1,2,6]. During energy depletion when cellular AMP levels are high relative to the adenosine triphosphate (ATP) concentration, AMPK activates energy-providing pathways including glucose uptake, glycolysis, or fatty acid oxidation [7,8,9,10]. Simultaneously, processes consuming ATP (e.g., gluconeogenesis, lipogenesis, or protein synthesis) are inhibited [7,8,9,10].

Being expressed in most mammalian cells, AMPK is a heterotrimeric protein consisting of a catalytic α (α1 or α2), scaffolding β (β1 or β2), and a regulatory nucleotide-binding γ (γ1, γ2, or γ3) subunit with the expression pattern differing from cell type to cell type [1,2,11,12,13,14]. Induction of AMPK activity involves phosphorylation of the conserved threonine residue Thr172 within the activation loop of the α subunit’s kinase domain by various protein kinases including the tumor suppressor liver kinase B1 (LKB1), Ca^2+^/calmodulin–dependent protein kinase kinase β (CaMKKβ), and transforming growth factor beta-activated kinase 1 [1,15,16,17,18,19,20,21,22,23,24,25,26,27,28]. AMPK activation in cellular energy depletion is primarily mediated by an increase in the AMP/ATP or ADP/ATP ratio [8,29,30]. Thus, AMP or ADP binding to the subunit at cystathionine-beta-synthase repeats results in conformational changes that allows for the phosphorylation at Thr172 by LKB1. This results in an enhancement of AMPK activity by >100-fold [1,8,12,15,31,32,33,34,35,36]. Moreover, AMP or ADP binding prevents dephosphorylation at Thr172 by protein phosphatases [8,12,37,38]. Additionally, binding of AMP, but not ADP, activates AMPK allosterically [8,11,12,37]. Conversely, ATP binding to the cystathionine-beta-synthase domain results in AMPK dephosphorylation by protein phosphatases [1,8,39].

Besides LKB1-associated regulation of AMPK phosphorylation, an alternative Ca^2+^-involving activation mechanisms independent of AMP exists [6,12,40,41]. Protein kinase CaMKKβ phosphorylates AMPK at Thr172 in response to elevated intracellular Ca^2+^ levels which may be caused by mediators such as thrombin or ghrelin [6,12,23,40,42,43]. Intracellular Ca^2+^ store depletion detected by the Ca^2+^-sensing protein stromal interacting molecule-1 leads to store-operated Ca^2+^ entry (SOCE) involving the Ca^2+^ release-activated Ca^2+^ channel Orai1 [44,45,46,47,48,49]. Orai1-mediated SOCE impacts on many cellular functions including cell proliferation, differentiation, migration, and cytokine production [44,50,51,52,53,54,55]. SOCE is involved in a sort of feedback mechanism involving AMPK: SOCE activates AMPK through CaMKKβ. AMPK in turn inhibits SOCE [45]. Moreover, AMPK inhibits SOCE by regulating Orai1 membrane abundance (at least in UMR106 cells) [44,56].

AMPK is a major regulator of whole body energy homeostasis [10,12], impacting on a variety of organs including liver [57,58,59,60,61], skeletal [62,63,64,65,66] and cardiac muscle [67,68,69,70,71,72,73], kidney [74,75,76,77], and bone [78,79,80]. In the kidney, AMPK regulates epithelial transport, podocyte function, blood pressure, epithelial-to-mesenchymal transition, autophagy as well as nitric oxide synthesis [75,76,81,82,83]. Not surprisingly, AMPK is highly relevant for renal pathophysiology, including ischemia, diabetic renal hypertrophy, polycystic kidney disease, chronic kidney disease, and hypertension [40,67,74,75,76]. This review summarizes the contribution of AMPK to the regulation of renal transport and hence to the final composition of excreted urine. Moreover, pathophysiological implications are discussed.

## 2. AMPK and Renal Tubular Transport

The kidney is particularly relevant for fluid, electrolyte, and acid–base homeostasis. In addition, it is an endocrine organ producing different hormones such as erythropoietin, Klotho, and calcitriol, the active form of vitamin D [84,85,86]. The kidneys are made up of about 1 million nephrons, their functional elements. A nephron comprises the glomerulus surrounded by the Bowman´s capsule, the proximal tubule, Henle’s loop, distal tubule, and the collecting duct. The primary urine is filtered in the glomerulus. Its composition is similar to plasma. In general, large molecules and particularly proteins >6000 Dalton are normally filtered to a low extent, if at all. The renal tubular system modifies the primary urine by reabsorbing or secreting ions and molecules, ultimately yielding the final urine [85,86,87]. Epithelial transport is mainly dependent on ATP-dependent pumps (primary-active), secondary-or tertiary-active transporters, as well as carriers and channels (passive, facilitated diffusion). Since active transport consumes energy by definition, it is not surprising that it is subject to regulation by AMPK. Moreover, even passive transport involving glucose transporter (GLUT) carriers is controlled by AMPK [74,75].

### 2.1. Na^+^/K^+^-ATPase

The ubiquitously expressed Na^+^/K^+^-ATPase is a primary active ATP-driven pump that mediates the basolateral extrusion of 3Na^+^ in exchange of 2K^+^, thereby establishing a transmembrane Na^+^ gradient, which is the prerequisite for secondary active Na^+^-dependent transport (e.g., through Na^+^-dependent glucose cotransporter 1 and 2 (SGLT1/2), Na^+^/H^+^ exchanger isoform 1 (NHE1), Na^+^-coupled phosphate transporter (NaPi-IIa), or Na^+^-K^+^-2Cl^−^ cotransporter (NKCC2), as discussed below) [75,88,89,90,91,92,93,94]. Almost one-third of the body’s energy is consumed by this pump [95]. Therefore, it does make sense that it is regulated by AMPK [74,75,76,94]: AMPK inhibits Na^+^/K^+^-ATPase in airway epithelial cells by promoting its endocytosis [96,97,98,99,100]. However, AMPK stimulates Na^+^/K^+^-ATPase membrane expression in skeletal muscle cells [101] and in renal epithelia [102], thereby counteracting renal ischemia-induced Na^+^/K^+^-ATPase endocytosis [103]. Interestingly, AMPKβ1 deficiency was found not to alter outcome in an ischemic kidney injury model in mice [104]. Hence, the effect of AMPK on Na^+^/K^+^-ATPase appears to be highly tissue-specific [74,75].

### 2.2. Proximal Tubule

A wide variety of luminal Na^+^-dependent cotransporters, which are secondary active, are involved in epithelial transport in the proximal tubule. Secondary active transporters utilize the energy of the transmembrane Na^+^ gradient generated by the primary active ATP-consuming Na^+^/K^+^-ATPase to facilitate transport of a substrate against its concentration gradient [105,106]. These transporters and the basolateral Na^+^/K^+^-ATPase consume substantial amounts of total cellular energy [74,75,107]. Hence, AMPK has been demonstrated to be an important regulator of proximal tubule transport [74,75].

#### 2.2.1. Glucose Transport

Since glucose is freely filtered by the glomerulus, glucose concentration in primary urine is similar to the plasma glucose concentration, whereas excreted urine is usually free of glucose [108,109,110]. The sugar is reabsorbed in the proximal tubule by the Na^+^-dependent glucose cotransporter 1 and 2 (SGLT1 and 2), the different expression patterns and properties of which ensure total glucose reabsorption as long as the plasma glucose concentration is not abnormally high [89,108]. SGLT2 has a high transport capacity but low affinity for glucose and is predominantly expressed in the kidney, while SGLT1 is also expressed in other tissues including the small intestine. SGLT2 contributes to the reabsorption of up to 90% of filtered glucose [108,109,111,112]. On the other hand, AMPK-regulated SGLT1 [7,92,113] has a low transport capacity but high affinity for glucose and reabsorbs the remaining glucose [108,109,110,114,115]. Glucose leaves the basolateral membrane through passive glucose carriers GLUT1 and GLUT2 [108,116,117,118]. AMPK activates SGLT1-dependent glucose transport, presumably by stimulating membrane insertion of the cotransporter as observed in colorectal Caco-2 cells [92,119]. In line with this, AMPK activation is associated with increased *SGLT1* expression and glucose uptake in cardiomyocytes [113,120]. Although the AMPK-dependent regulation of SGLT1 in the proximal tubule has not explicitly been addressed, it is tempting to speculate that it is similar to other cell types [92,113,119,120]. The regulation of SGLT by AMPK is a doubled-edged sword: on the one hand, SGLT1-dependent reabsorption of glucose in proximal tubular cells requires energy which is generated by β-oxidation of fatty acids to a large extent [121,122]. On the other hand, it prevents the loss of energy-rich glucose [122,123], thereby maintaining the Na^+^/K^+^-ATPase-facilitated Na^+^ gradient for Na^+^-dependent transport and many other cellular processes [75,76]. SGLT1-mediated glucose uptake is linked to the GLUT1-dependent efflux at the basolateral side [108,116]. GLUT1 activity is stimulated by AMPK in various cell types [124,125,126,127,128,129,130,131]. Therefore, it is conceivable that renal GLUT1 might also be regulated by AMPK in order to save energy-providing glucose. In line with this, Baldwin et al. (1997) showed enhanced glucose uptake via GLUT1 in baby hamster kidney cells treated with AMPK activator 5-aminoimidazole-4-carboxamide ribonucleotide (AICAR) [132]. Moreover, Sokolovska et al. (2010) reported that metformin, another pharmacological AMPK activator, increased *GLUT1* gene expression in rat kidneys [133]. Also, AMPK activation was associated with enhanced activity of GLUT2. These studies, however, found reduced SGLT1 membrane abundance upon AMPK activation, at least in the case of murine intestinal tissue [134,135].

#### 2.2.2. Na^+^/H^+^ Exchanger Isoform 1

The ubiquitous Na^+^/H^+^ exchanger isoform 1 (NHE1) participates in cell volume and pH regulation by extruding one cytosolic H^+^ in exchange for one extracellular Na^+^ [136,137]. NHE1 is expressed in all parts of the nephron, including the proximal tubule. However, it cannot be detected in the macula densa and intercalated cells of the distal nephron [136,138,139]. In the proximal tubule, NHE1 is particularly important for HCO_3_^−^ reabsorption [140]. In hypoxia, anaerobic glycolysis is predominant, which results in intracellular accumulation of lactate and H^+^ [90]. Acidosis, however, inhibits glycolysis [90,141,142] and would jeopardize cellular energy generation. AMPK-dependent stimulation of NHE1 activity in human embryonic kidney (HEK) cells therefore helps cells keep up anaerobic glycolysis in oxygen deficiency, as demonstrated by Rotte et al. (2010) [90]. Given that NHE1 is needed for proximal tubular HCO_3_^−^ reabsorption [140], AMPK may help retain HCO_3_^−^, thereby alleviating acidosis in energy deficiency and hypoxia.

#### 2.2.3. Creatine Transporter

In some organs with high metabolic activity, including skeletal muscle, heart, and brain, creatine is used to refuel cellular ATP levels [143,144,145]. In the proximal tubule, creatine, a small molecule that is freely filtered, is also reabsorbed through secondary active Na^+^-dependent creatine transporter (CRT) (SLC6A8) [7,75,143,146]. AMPK has been demonstrated to downregulate CRT activity and apical membrane expression in a polarized mouse S3 proximal tubule cell line, presumably through mammalian target of rapamycin signaling [147]. The AMPK-dependent inhibition of CRT may help reduce unnecessary energy expenditure [75]. Conversely, AMPK stimulates CRT-mediated creatine transport in cardiomyocytes [148,149]. This again demonstrates that AMPK effects are tissue-specific [148].

#### 2.2.4. Na^+^-Coupled Phosphate Transporter IIa

Inorganic phosphate is mainly reabsorbed by the secondary active Na^+^-coupled phosphate transporter (NaPi-IIa) (SLC34A1) in the proximal tubule [93,150,151,152]. Employing electrophysiological recordings in *Xenopus* oocytes, it was shown that AMPK inhibits NaPi-IIa [93]. Kinetics analysis revealed that AMPK decreases NaPi-IIa membrane expression rather than changing its properties.

The regulation of phosphate metabolism by AMPK is not restricted to NaPi-IIa: Recently, AMPK was demonstrated to control the formation of bone-derived hormone fibroblast growth factor 23 (FGF23) [56], which induces renal phosphate excretion by extracellular-signal regulated kinases 1/2 (ERK1/2)-mediated degradation of membrane NaPi-IIa [150]. AMPK inhibits FGF23 production in cell culture and in mice [56]. Despite markedly elevated FGF23 serum levels in AMPKα1-deficient mice, renal phosphate excretion was not different from wild-type animals [56]. The same holds true for cellular localization of NaPi-IIa and renal ERK1/2 [56]. Thus, it is possible that AMPK deficiency is paralleled with some FGF23 resistance.

### 2.3. Loop of Henle

#### 2.3.1. Na^+^-K^+^-2Cl^−^ Cotransporter

The Na^+^-K^+^-2Cl^−^ cotransporter (NKCC2), expressed in the thick ascending limb (TAL) of the loop of Henle and macula densa, is required for the generation of a hypertonic medullary interstitium, a mechanism needed for concentrating urine [75,76,88,91]. NKCC2 is a direct substrate of AMPK which phosphorylates it at its stimulatory serine residue Ser-126 [153]. Moreover, exposure of murine macula densa-like cells to low salt leads to AMPK activation and increased NKCC2 phosphorylation [154]. In addition, increased subapical expression (and apparent reduced apical expression) of NKCC2 in the medullary TAL of the loop of Henle along with elevated urinary Na^+^ excretion in AMPKβ1-deficient mice on a normal salt diet were observed [155]. This is in line with AMPK being an important regulator of NKCC2-mediated salt retention in the medullary TAL of Henle [155]. Efe et al. (2016) recently observed markedly increased outer medullary expression of NKCC2 in rats treated with the AMPK activator metformin [156]. However, according to a recent in vivo study by Udwan et al. (2017), a low salt diet induced upregulation of NKCC2 surface expression in mouse kidneys but left AMPK activity unchanged [157]. Therefore, the exact role of AMPK in stimulating NKCC2 remains to be established.

#### 2.3.2. Renal Outer Medullary K^+^ Channel

The apical renal outer medullary K^+^ channel (ROMK) is required for NKCC2 to work properly, as it allows the recirculation of K^+^ ions taken up by NKCC2 into the lumen [75,88]. AMPK is an inhibitor of ROMK by downregulating both channel activity and membrane abundance of the channel protein in a heterologous expression system using *Xenopus* oocytes [158]. In vivo studies revealed that the AMPK effect on ROMK is relevant for the renal excretion of K^+^ after an acute K^+^ challenge, as upregulation of renal ROMK1 protein expression and the ability of K^+^ elimination were more pronounced in AMPKα1-deficient than in wild-type mice [158].

### 2.4. Distal Tubule

#### 2.4.1. Cystic Fibrosis Transmembrane Conductance Regulator

The ATP-gated and cyclic AMP (cAMP)-dependent Cl^−^ channel cystic fibrosis transmembrane conductance regulator (CFTR) participates in Cl^−^ secretion and is broadly known for its role in cystic fibrosis, the pathophysiology of which is due to channel malfunction [74,75,76,159]. In the kidney, CFTR contributes to Cl^−^ secretion in the distal tubule and the principal cells of the cortical and medullary collecting ducts [74,75,160]. AMPK has been demonstrated to inhibit CFTR-dependent Cl^−^ conductance in *Xenopus* oocytes [159] and to decrease CFTR channel activity in the lung [161,162] and colon [163]. cAMP-stimulated cell proliferation and CFTR-dependent Cl^−^ secretion play a decisive role for epithelial cyst enlargement in autosomal dominant polycystic kidney disease (ADPKD) [164]. In line with this, AMPK activation inhibits CFTR in Madin-Darby canine kidney (MDCK) cells [165] as well as decreases cystogenesis in murine models of ADPKD [165,166], suggesting a potential role for pharmacological AMPK activation in the treatment of ADPKD [165,166].

#### 2.4.2. Ca^2+^ Transport

Most Ca^2+^ is reabsorbed by passive paracellular diffusion along with other ions and water through tight junctions in the proximal tubule and the more distal parts of the nephron [88,167]. Conversely, only 5–10% of filtered Ca^2+^ is reabsorbed by transcellular transport involving the apical transient receptor potential vanilloid 5 channel TRPV5 in the distal convoluted tubule [88]: Ca^2+^ enters the cell through TRPV5, whereas basolateral Ca^2+^ efflux is accomplished by the Na^+^/Ca^2+^ exchanger (NCX) and the Ca^2+^-ATPase [88,167,168]. AMPK has been shown to inhibit NCX and decrease Orai1-mediated SOCE in murine dendritic cells [169]. Therefore, it is tempting to speculate that Ca^2+^ reabsorption may be downregulated in the distal tubule in ATP deficiency [169,170]. Indeed, AMPK downregulates Orai1-dependent SOCE in T-lymphocytes [171], endothelial cells [45], and in osteoblast-like cells [56]. Since renal Orai1 activity contributes to kidney fibrosis [172], AMPK-mediated Orai1 downregulation may also be therapeutically desirable.

### 2.5. Collecting Duct

#### 2.5.1. Epithelial Na^+^ Channel

In the collecting duct, fine tuning of Na^+^ and K^+^ homeostasis is accomplished by epithelial Na^+^ channel (ENaC) and ROMK K^+^ channel. Both channels are controlled by the renin-angiotensin-aldosterone system [173,174,175] regulating extracellular volume and hence arterial blood pressure [173,174,175,176,177]. Na^+^ reabsorption by ENaC in the late distal convoluted tubule and cortical collecting duct principal cells is a highly energy-demanding process, as it utilizes the electrochemical driving force generated by the basolateral Na^+^/K^+^-ATPase [74,75,76,176,178]. AMPK inhibits epithelial Na^+^ transport in various tissues, including lung [96,179], colonic [180], and renal cortical collecting duct cells [180,181,182,183]. In line with this, AMPKα1-deficient mice exhibit increased renal ENaC expression [180]. In detail, AMPK downregulates ENaC surface expression by inducing the binding of the ubiquitin ligase neural precursor cell expressed developmentally downregulated protein 4-2 (Nedd4-2) to ENaC subunits, resulting in ENaC ubiquitination with subsequent endocytosis and degradation [177,180,184]. In line with this, activation of AMPK enhances the tubuloglomerular feedback and induces urinary diuresis and Na^+^ excretion in rats [185]. However, AMPKα1^−/−^ mice with genetic kidney-specific AMPKα2 deletion exhibit a moderate increase in diuresis and natriuresis, possibly because NKCC2 activity is insufficient despite upregulated ENaC activity [186]. Taken together, AMPK activity limits ENaC-dependent energy-consuming Na^+^ reabsorption [177,180,181,185].

#### 2.5.2. Voltage-Gated K^+^ Channel

The voltage-gated K^+^ channel (KCNQ1) is important for the cardiovascular system as well as for electrolyte and fluid homeostasis and is expressed in the distal nephron including the collecting duct [170,187,188,189]. Its exact role is ill-defined, although a contribution to cell volume regulation is postulated [75,187]. Similar to ENaC, AMPK inhibits KCNQ1 via Nedd4-2, as demonstrated in collecting duct principal cells of rat ex vivo kidney slices [187], MDCK cells [190], and *Xenopus* oocytes [191].

#### 2.5.3. Vacuolar H^+^-ATPase

The primary active vacuolar H^+^-ATPase (V-ATPase) is located at the apical membrane of proximal tubule cells and collecting duct type A intercalated cells. It contributes to the regulation of acid–base homeostasis by secreting H^+^ ions into the tubular lumen [76,192,193]. AMPK inhibits the protein kinase A (PKA)-dependent membrane expression of V-ATPase in collecting duct intercalated cells of rat ex vivo kidney slices [193]. Moreover, epididymal proton-secreting clear cells, developmentally related to intercalated cells, exhibit reduced apical membrane abundance of V-ATPase after in vivo perfusion with the AMPK activator 5-aminoimidazole-4-carboxamide-1-beta-D-ribofuranoside (AICAR) into rats [194]. It appears to be likely that energy deficiency limits highly energy-consuming primary active H^+^ excretion in the proximal tubule, whereas secondary active NHE1-dependent H^+^ secretion is maintained, thereby keeping up at least anaerobic glycolysis [192]. The opposing effects of AMPK and PKA on V-ATPase expression and activity in kidney intercalated cells can be explained by different phosphorylation sites, as AMPK and PKA phosphorylate the A subunit at Ser-384 and Ser-175, respectively [195,196]. McGuire and Forgac (2018) further demonstrated that AMPK increases lysosomal V-ATPase assembly and activity in HEK293T cells under conditions of energy depletion [197]. In cells depleted of energy, acidification of autophagic intracellular compartments by V-ATPases enables the lysosomal degradation of proteins and lipids to generate energy substrates for ATP production [197,198]. Thus, it appears to be likely that AMPK-regulated V-ATPase activity depends on its concrete cellular localization and function [197].

#### 2.5.4. Water and Urea Handling

AMPK also regulates renal urea and water handling [76,199]. In the inner medullary collecting duct, osmotic gradients are generated by NKCC2 and urea transporter UT-A1 and water is reabsorbed through aquaporin 2 (AQP2) [76,156,199,200]. The concentration of urine requires the antidiuretic hormone vasopressin, which binds to vasopressin type 2 receptors of collecting duct principal cells, resulting in cAMP-mediated activation of PKA and subsequent phosphorylation and apical membrane insertion of AQP2 and UT-A1 [76,156,199]. Congenital nephrogenic diabetes insipidus (NDI) is a disease primarily caused by mutations of vasopressin type 2 receptors that is characterized by renal resistance to vasopressin and limited urine concentrating capacity [156,201]. According to two in vivo studies using rodent models of congenital NDI, the metformin-stimulated AMPK activation ameliorates the ability of the kidney to concentrate urine by increasing the phosphorylation and apical membrane expression of inner medullary AQP2 and UT-A1 [156,202]. In contrast, an ex vivo treatment of rat kidney slices with AICAR led to reduced apical membrane insertion of AQP2 [203]. Moreover, AMPK antagonizes the desmopressin-induced AQP2 phosphorylation in vitro, thus also suggesting an inhibitory function of AMPK on AQP2 regulation [203]. It appears likely that AMPK-independent effects of the pharmacological AMPK agonists contribute to this discrepancy [156,202,203]. Thus, further studies are clearly required.

## 3. Conclusions and Perspectives

**Figure 1:**
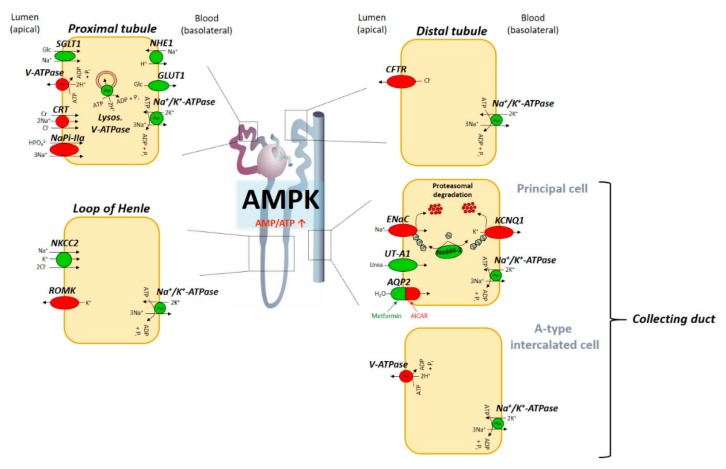
Tentative model illustrating AMPK-dependent effects on renal transport along the nephron. Cellular energy depletion (e.g., during hypoxia) leads to an elevated AMP/ATP ratio and subsequent AMPK activation. AMPK in turn regulates a multitude of active and passive epithelial transport processes along the renal tubular system in order to maintain cellular energy homeostasis. Ion channels, transport proteins, and ATPases that are activated upon AMPK stimulation are depicted as green icons, whereas red coloring indicates AMPK-dependent inhibition (see text for details). AMP, 5’-adenosine monophosphate; AMPK, AMP-activated protein kinase; SGLT1, Na^+^-dependent glucose cotransporter 1; V-ATPase, vacuolar H^+^-ATPase; CRT, creatine transporter; NaPi-IIa, Na^+^-coupled phosphate transporter IIa; NHE1, Na^+^/H^+^ exchanger isoform 1; GLUT1, glucose transporter 1; NKCC2, Na^+^-K^+^-2Cl^−^ cotransporter; ROMK, renal outer medullary K^+^ channel; CFTR, cystic fibrosis transmembrane conductance regulator; ENaC, epithelial Na^+^ channel; KCNQ1, voltage-gated K^+^ channel; Nedd4-2, neural precursor cell expressed developmentally downregulated protein 4-2; UT-A1, urea transporter A1; AQP2, aquaporin 2.

A growing list of studies indicates the pivotal role of AMPK as a metabolic-sensing regulator of a multitude of transport processes in the kidney [7,74,75,76,170]. Particularly, AMPK activation under conditions of energy deficiency is expected to differentially modulate renal epithelial ion transport in order to preserve cellular energy homeostasis (Figure 1) [7,74,75,76,94,170]. Alongside the above discussed function of AMPK in kidney tubular transport, a variety of other transport proteins, which are expressed in the kidney as well, are regulated by AMPK in extrarenal tissues [7,94,170,204] that are reviewed elsewhere [170] and [7] and summarized in Table 1. Future studies are required to focus on the therapeutic value of pharmacological AMPK manipulation to combat kidney disease [74,75,76,205,206].

## Figures and Tables

**Table 1:** Overview of transport proteins regulated by AMPK in extrarenal tissues and evidence for renal expression.

Ion Channel/Transporter and Method of Modifying AMPK Activity	AMPK Effect	Cell Type of Studied AMPK Effect/Ref.	Evidence for Renal Expression/Ref.
**Heterologous expression systems**			
**Kir2.1**	Reduction of channel activity and membrane abundance via Nedd4-2 mediated endocytosis	*Xenopus* oocytes [207]	Human proximal tubular cells [208]
**Kv1.5**	Reduction of channel activity and membrane abundance via Nedd4-2 mediated endocytosis	*Xenopus* oocytes [209]	Human kidney biopsies [210]
**Kv11.1 (hERG)**	Reduction of channel activity and membrane abundance via Nedd4-2 mediated endocytosis	*Xenopus* oocytes [211]	Human proximal and distal convoluted tubule [212]
**SMIT**	Reduction of channel activity	*Xenopus* oocytes [213]	Rat kidney medulla [214]
**BGT1**	Reduction of channel activity	*Xenopus* oocytes [213]	Human kidney inner medulla [215] and mouse kidney medulla (basolateral membranes of collecting ducts and TAL of Henle) [216]
**EAAT3**	Reduction of channel activity and membrane abundance	*Xenopus* oocytes [217]	Mouse renal proximal tubule [218]
**NCX**	Reduction of channel activity and membrane abundance	*Xenopus* oocytes [169]	Rat distal convoluted tubule [219]
**K_2P_10.1 (TREK-2)**	Inhibition of channel activity via phosphorylation at Ser-326 and Ser-359	HEK293 cells [220]	Human proximal tubule [221]
**K_Ca_1.1**	Increase in channel activity and membrane abundance	*Xenopus* oocytes [222]	Human clear cell renal cell carcinoma (ccRCC) and healthy kidney cortex [223]
**Pharmacological Manipulation**			
**K_Ca_1.1**	Inhibition of channel activity	Rat carotid body type I cells [224]	
**Kir6.2**	Upregulation of channel activity Up- or down-regulation of channel activity	Rat cardiomyocytes [225] Rat pancreatic beta-cells [226,227]	Rat renal tubular epithelial cells [228]
**KCa3.1**	Reduction of channel activity	Human airway epithelial cells [229]	Human proximal tubular cells [230]
**MCT1 and MCT4**	Upregulation of mRNA expression	Rat skeletal muscle [231]	MCT1: basolateral membrane of mouse proximal tubular epithelial cells [232] MCT4: human ccRCC [233]
**PepT1**	Downregulation of channel activity and brush-border membrane abundance	Caco-2 cells [234]	Rat renal proximal tubule [235]
**Orai1**	Downregulation of cell membrane abundance and SOCE	Rat UMR106 osteoblast-like cells [56]	Rat glomerular mesangial cells [236]
**Genetically Modified Mouse Models**			
**Orai1**		Mouse T-lymphocytes [171] Mouse dendritic cells [169]	

## Abbreviations

ADP	Adenosine diphosphate
ADPKD	Autosomal dominant polycystic kidney disease
AMPK	5′-adenosine monophosphate (AMP)–activated protein kinase
AQP2	Aquaporin 2
ATP	Adenosine triphosphate
BGT1	Betaine/γ-aminobutyric acid (GABA) transporter 1
CaMKKβ	Ca^2+^/calmodulin–dependent protein kinase kinase β
cAMP	Cyclic adenosine monophosphate
ccRCC	Clear cell renal cell carcinoma
CFTR	Cystic fibrosis transmembrane conductance regulator
CRT	Creatine transporter
EAAT3	Excitatory amino acid transporter 3
ENaC	Epithelial Na^+^ channel
ERK1/2	Extracellular-signal regulated kinases 1/2
FGF23	Fibroblast growth factor 23
GLUT	Glucose transporter
HEK	Human embryonic kidney cells
hERG	Human ether-a-go-go-related gene
Kca	Ca^2+^ activated K^+^ channels
KCNQ1	Voltage-gated K^+^ channel
Kir	Inwardly rectifying K^+^ channels
Kv	Voltage gated K^+^ channels
LKB1	Liver kinase B1
MCT	Monocarboxylate transporters
MDCK	Madin-Darby canine kidney cells
NaPi-IIa	Na^+^-coupled phosphate transporter
NCX	Na^+^/Ca^2+^ exchanger
NDI	Nephrogenic diabetes insipidus
Nedd4-2	Neural precursor cell expressed developmentally down-regulated protein 4-2
NHE1	Na^+^/H^+^ exchanger isoform 1
NKCC2	Na^+^-K^+^-2Cl^−^ cotransporter
PepT1	H^+^-coupled di- and tripeptide transporter 1
PKA	Protein kinase A
ROMK	Renal outer medullary K^+^ channel
SGLT	Na^+^-dependent glucose cotransporter
SMIT	Na^+^ coupled myoinositol transporter
SOCE	Store-operated Ca^2+^ entry
TAL	Thick ascending limb
TREK-2	Tandem pore domain K^+^ channel 2
TRPV5	Transient receptor potential vanilloid 5 channel
UT	Urea transporter
V-ATPase	Vacuolar H^+^-ATPase
